# Effective Downsizing of a Large Oesophageal Gastrointestinal Stromal Tumour with Neoadjuvant Imatinib Enabling an Uncomplicated and without Tumour Rupture Laparoscopic-Assisted Ivor-Lewis Oesophagectomy

**DOI:** 10.1155/2015/165736

**Published:** 2015-05-05

**Authors:** Kyriakos Neofytou, Mafalda Costa Neves, Alexandros Giakoustidis, Charlotte Benson, Satvinder Mudan

**Affiliations:** ^1^Royal Marsden Hospital, Department of Academic Surgery, Upper GI/HPB Unit, Fulham Road, London SW3 6JJ, UK; ^2^Royal Marsden Hospital, Sarcoma Unit, Fulham Road, London SW3 6JJ, UK

## Abstract

Neoadjuvant imatinib for gastrointestinal stromal tumours (GISTs) is increasingly used nowadays. As oesophagectomy is associated with high morbidity and mortality, a preoperative downsizing of an oesophageal GIST to limit the extent of resection would be ideal. Because these tumours are rare and neoadjuvant treatment with imatinib is recent, there is limited literature available regarding neoadjuvant administration of imatinib in patients with oesophageal GISTs. A 50-year-old woman presented with total dysphagia. An upper endoscopy and biopsy revealed a large submucosal KIT-positive GIST obstructing the mid oesophagus. CT confirmed a lesion measuring 99 mm × 50 mm × 104 mm. Because the size and location of the tumour increased the risk of intraoperative rupture, it was decided to administer preoperative imatinib. The patient had an excellent clinical and radiological response. Her dysphagia gradually resolved and the follow-up CT scans of the first 10 months showed a gradually reducing tumour size to 54 mm × 33 mm × 42 mm. The patient underwent an uneventful laparoscopic-assisted Ivor-Lewis oesophagectomy. Postoperatively, the patient continued with adjuvant imatinib. At the last follow-up, 1 year from operation and 38 months from the diagnosis, the patient is disease free.

## 1. Introduction

Gastrointestinal Stromal Tumours (GISTs) are mesenchymal neoplasms located primarily in the gastrointestinal (GI) tract and represent 80% of all GI sarcomas [1]. Surgery is the only potentially curative option, and for approximately 60% of patients, the successful surgical removal of the tumour would represent cure [2].

A benchmark in dealing with these chemotherapy-insensitive tumours was the introduction of treatment with imatinib and later of sunitinib, two tyrosine-kinase inhibitors [3, 4]. The indications for imatinib use are locally advanced inoperable disease, metastatic disease, and adjuvant therapy in patients at high risk for relapse [3].

Although 80% of newly diagnosed cases are resectable at presentation, the high efficacy of imatinib in the metastatic and adjuvant setting quickly made way for its usage also in the neoadjuvant setting [4–6].

The main indications for preoperative imatinib are (1) unresectable or metastatic disease with an aim of tumour downsizing and conversion of patient to candidate for radical resection and (2) resectable metastatic or nonmetastatic disease with an aim of downsizing of disease as well, thus reducing the extent of surgery and postoperative morbidity and mortality (e.g., laparoscopic removal avoiding the need for multivisceral resection, local excision versus more radical interventions for tumours in difficult anatomical locations such as the rectum, duodenum, and oesophagus). In this second group of patients, with resectable disease at presentation, preoperative administration of imatinib also aims to improve the oncological outcome by reducing the likelihood of intraoperative tumour rupture, which has been identified as being an independent predictor of disease recurrence and is currently included in systems assessing the likelihood of postoperative relapse [2, 7].

Oesophageal GISTs are very rare, representing less than 2% of GISTs [8]. Because of the high morbidity/mortality associated with oesophagectomy, preoperative downsizing of oesophageal GIST is ideal, in an attempt to reduce the extent of surgery and consequently the risk of perioperative complications. Due to the rarity of these tumours and the fact that neoadjuvant treatment with imatinib is a recent development, there is limited literature available regarding neoadjuvant administration of imatinib in patients with oesophageal GISTs [9–12].

We report a case of large oesophageal GIST in a 50-year-old woman who underwent laparoscopic-assisted Ivor-Lewis oesophagectomy after 26 months of neoadjuvant imatinib therapy.

## 2. Case Report

A 50-year-old lady presented to our hospital with a 4-month history of progressive dysphagia. Her symptoms worsened progressively resulting in total dysphagia, pain mainly in the epigastric and lower chest area radiating to her back, and one recent episode of haematemesis. At presentation, she was unable to swallow even her saliva. She had no associated medical comorbidities, except for being an ex-smoker and having a BMI of 41. The physical examination was unremarkable. Standard laboratory test results were within normal range, with haemoglobin of 11 mg/dL. An oesophagogastroscopy revealed a large submucosal obstructing oesophageal mass, partially ulcerated, situated 42 mm above diaphragmatic hiatus, and extending over a distance of at least 10 cm. Biopsy revealed a GIST with diffuse positivity for CD117 and CD34 and a mitotic index of 3/22 hpf (insufficient material to count mitoses/50 fields). Gene analysis with direct sequencing revealed a 6 bp deletion in exon 11 of the KIT gene [mutation details: c.1670_1675del p.Trp557_Val559delinsPhe (reference cDNA sequence: NM_000222.2)].

**Figure 1:**
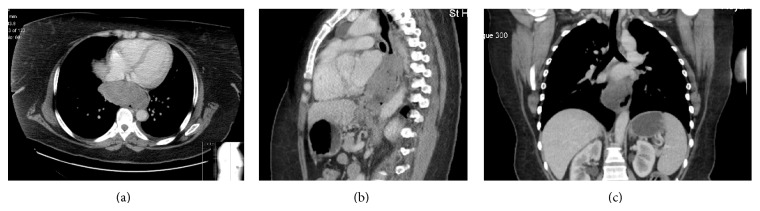
CT scan at presentation: (a) axial view, (b) sagittal view, and (c) coronal view.

A computed tomography (CT) scan of chest and abdomen revealed an annular polypoidal mass, 99 mm × 50 mm × 104 mm in diameter in the mid oesophagus, arising 42 mm above the diaphragmatic hiatus and extending cranially over a distance of 10 cm to the subcarinal level (Figure 1). There was evidence of extension through the oesophageal wall over a distance of at least 10 mm but without lymphadenopathy or metastatic disease. The tumour was classified as high risk according to Fletcher's classification [13].

The patient was admitted to our hospital for pain and symptoms of total dysphagia and an 8F nasogastric tube was inserted endoscopically.

Her case was further discussed in the sarcoma Multidisciplinary Team (MDT) meeting. Despite the tumour being resectable at presentation, its size and location rendered it to an increased risk of intraoperative rupture. Furthermore, GIST with mutations in exon 11 of KIT is generally associated with sensitivity to imatinib. Therefore, the MDT decided on preoperative administration of imatinib 400 mg daily.

She had an excellent clinical response with dysphagia gradually improving, and 12 days later, she was able to tolerate solid food once again.

**Figure 2:**
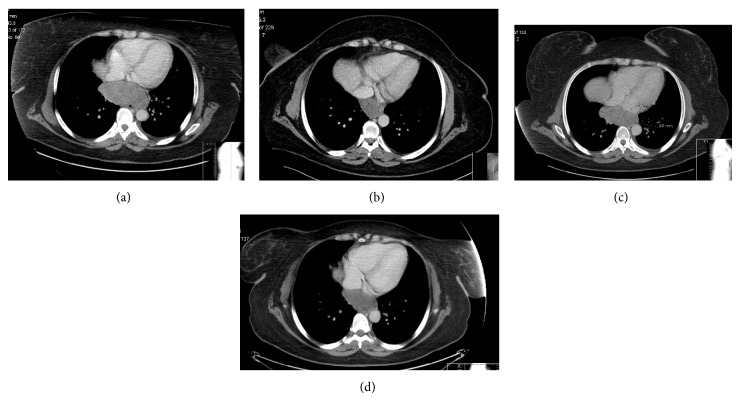
(a) CT scan at diagnosis, Longest Axial Diameter (LAD) 99 mm; (b) CT scan after 10 months of imatinib therapy, partial response LAD 54 mm; (c) CT scan after 13 months of imatinib therapy, disease progression LAD 90 mm; and (d) CT scan after 26 months of imatinib therapy, partial response LAD 44 mm.

**Figure 3:**
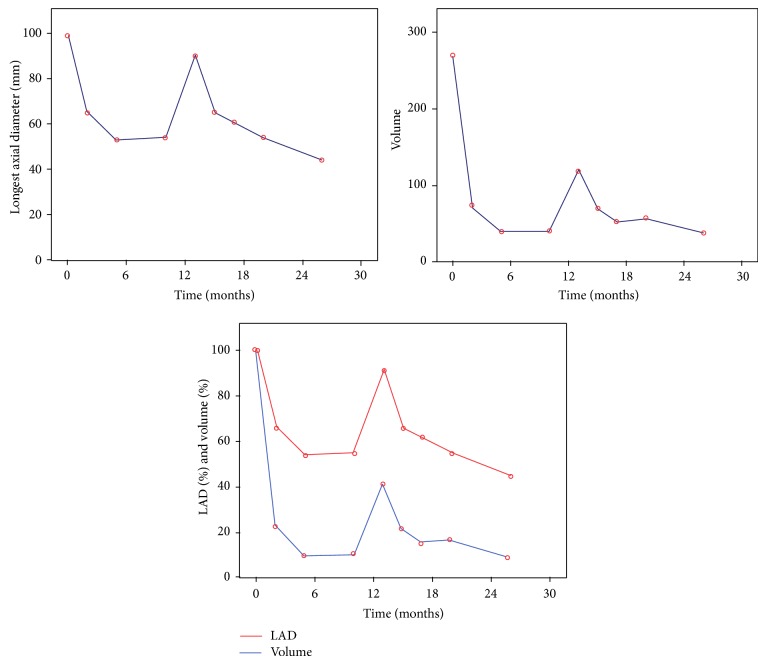
Changes of Longest Axial Diameter and volume (cm^3^) during imatinib therapy.

The first follow-up CT scan two months after initiating treatment showed an excellent partial response, with the mass measuring 65 mm × 42 mm × 51 mm and reduction of tumour mass to 27% of the initial volume. New CT three months after showed further response, with mass reduction to 53 mm × 33 mm × 42 mm (Figures 2 and 3). During this period, she had been tolerating treatment very well, apart from Grade 1 fatigue.

After ten months of treatment, the tumour had been stabilized at 54 mm × 33 mm × 42 mm, suggesting plateau response. According to our policy, this would be the correct timing for surgery. However, because the patient was considered high risk for operation, as indicated by her cardiopulmonary exercise testing (CPX) revealing an anaerobic threshold of 9.3, the MDT decided the patient should continue treatment with imatinib and further take up an exercise program in an attempt to reduce her risks of perioperative complications.

Unfortunately, the next CT scan after 13 months of treatment demonstrated localized but progressive disease (90 mm × 42 mm × 60 mm) (Figures 2 and 3). Although disease progression under treatment with imatinib is a well described phenomenon, the previous excellent response in combination with the very fast disease progression raised the question of drug compliance [6]. On further questioning, the patient admitted not being compliant with drug therapy because of side effects of dry skin and periorbital oedema.

Further management of this patient was challenging. On one hand, we were not keen to lose the surgical window of opportunity for a bulky but localised tumour of the oesophagus, but on the other hand, the risk of intraoperative tumour rupture was similar to that prior to treatment.

After extensive discussion with the patient regarding the advantages and disadvantages of continuing imatinib therapy, the patient agreed to restart treatment.

She received her treatment regularly, and the follow-up CTs during the next year showed progressive reduction of the tumour size (Figures 2 and 3). 26 months after diagnosis, and with the latest CT demonstrating the oesophageal tumour measuring 44 mm × 36 mm × 35 mm, equal to 13.84% of the volume at diagnosis, the patient underwent an uncomplicated and without tumour rupture laparoscopic-assisted Ivor-Lewis oesophagectomy and was discharged 12 days after surgery.

Histopathology revealed a GIST with extensive areas of hypocellularity with hyalinised stroma and haemosiderin deposition, consistent with therapy effect, and viable tumour was estimated at approximately 30–50%. There was mucosal ulceration and the lesion extended from the mucosa to the adventitia. The tumour margins measured 10 mm from the oesophageal resection margin, >30 mm from gastric resection margin, and <1 mm from the serosal surface. Nine lymph nodes were identified, with no evidence of metastatic tumour. The mitotic index was 14/50 hpf.

Both the original size of the tumour and the mitotic index (14/50 hpf) classified the tumour as “high risk” and the decision was taken for further adjuvant imatinib, according to the Scandinavian Sarcoma Group Trial [14].

At the last follow-up, 1 year after operation and 38 months from the diagnosis, the patient remained disease free.

## 3. Discussion

We present a patient with a large oesophageal GIST who underwent radical surgical resection following neoadjuvant administration of imatinib for 26 months. Despite the fact that the plateau response to imatinib was reached 10 months after initiating treatment, the combination of high operative risk and noncompliance with therapy prolonged the neoadjuvant administration of imatinib. The lack of compliance to daily dosage imatinib resulted in the rapid enlargement of the tumour. The subsequent improved drug compliance resulted in a new response to treatment, with a better radiological response than the one seen on plateau phase before. Finally, the patient underwent an uneventful laparoscopic-assisted Ivor-Lewis oesophagectomy without tumour rupture. Postoperatively, the patient continued imatinib treatment and remained disease free at 1 year follow-up.

The discovery that the vast majority of GISTs have characteristic activating mutations in c-KIT (75–80%) or PDGFRA (5–15%) radically changed the management of these tumours by the approval of the use of tyrosine-kinase inhibitors [4, 15]. Imatinib, the first approved drug in this category, is an adenosine triphosphate, competitive inhibitor of KIT, PDGFRA, BCR-ABL, and ABL tyrosine kinases, which was already used in the treatment of patients with chronic myeloid leukemia (CML) who bear the BCR-ABL fusion gene [3, 16]. The approval of sunitinib followed, which is another inhibitor of several tyrosine kinases, including KIT, PDGFR, and VEGFR for the treatment of patients with imatinib-resistant GIST or patients who do not tolerate imatinib [4].

Although preoperative downsizing of oesophageal GIST in an attempt to reduce the high morbidity/mortality associated with oesophagectomy seems ideal, literature available regarding neoadjuvant administration of imatinib in patients with oesophageal GISTs is very limited as oesophageal GISTs are rare and also neoadjuvant treatment with imatinib is a recent development [9–12]. Yanagawa et al. reported the successful downsizing of a large GIST of lower oesophagus after 6 months of neoadjuvant imatinib administration, allowing for a complete resection of the tumour without rupture [9]. Tirumani et al., in a recent study regarding the timing of earliest, best, and plateau response to neoadjuvant imatinib in patients with GIST, reported that one of the 20 patients who were enrolled in this study was diagnosed with oesophageal GIST and underwent Ivor-Lewis oesophagectomy after 20 weeks of neoadjuvant administration of imatinib with stable disease [10]. Fiore et al. studied the efficacy of preoperative administration of imatinib for unresectable or locally advanced primary GIST, and the authors included in their study one patient with locally advanced oesophageal GIST (9 cm) who had partial response according to RECIST after 9 months of imatinib administration and who then underwent an uncomplicated Ivor-Lewis oesophagectomy [11]. Shinagare et al. have also reported a patient with oesophageal GIST who underwent esophagectomy after 4 months of neoadjuvant administration of imatinib [12]. As we can see from the above mentioned cases, the experience regarding the preoperative administration of imatinib in patients with oesophageal GIST is limited, and experience from dealing with such cases could give way to answering questions regarding the benefit of patients related to the oncological outcome and also the optimal duration of the preoperative administration of imatinib.

The accurate prediction of recurrence is very important for the decision of administration of adjuvant imatinib. The existing risk stratification systems (Fletcher's classification, Joensuu liner-model, AFIP criteria, and modified NIH criteria) are based on tumour size, mitotic activity, tumour rupture prior to or at surgery, and tumour location [2, 7, 13, 17].

Regarding oesophageal GISTs, despite the fact that these can be classified according to the risk stratification systems mentioned above, the contribution of oesophageal GISTs to the creation of these systems was very small. This fact raises concerns about the accuracy of these systems regarding the prognosis of patients with oesophageal GISTs. For example, only 8 of the 1625 patients (0.49%) who validated for the creation of Joensuu liner-model had oesophageal GIST [2]. Also, it is worth noting that none of these systems include patients who received neoadjuvant imatinib. A similar lack of information is observed regarding the effectiveness of adjuvant administration of imatinib in patients with oesophageal GISTs. Although Joensuu et al. demonstrated the superiority of administration of adjuvant imatinib for 3 years when compared to 1 year with regard to disease free and overall survival for patients with KIT-positive GISTs, once again the number of patients with oesophageal GISTs included in this study was very small [14].

The optimal duration of preoperative imatinib administration is not fully documented. NCCN states that the treatment should be continued until the tumour stops responding to imatinib or if PD appears unresponsive even to increased dose of imatinib [18]. Interruption of preoperative imatinib despite continuous tumour response is also acceptable, with the assumption that any further reduction in volume will not affect the type and extent of surgery [6, 10]. There are published studies about the duration of preoperative administration of imatinib ranging from a few days to more than one year [6, 19, 20].

Disease progression during neoadjuvant imatinib has been reported as a rare phenomenon for primary tumours, which has been attributed in part to the emergence of secondary mutations following prolonged treatment [6, 21]. Moreover, the increased understanding of mutational status with respect to KIT and pDGFRA mutations has meant that patients with truly imatinib insensitive disease (e.g., PDGFRA D842V mutations) are not considered for treatment. The hypothesis of the emergence of secondary mutations was considered when our patient demonstrated progressive disease after 13 months of imatinib. However, the explanation was much simpler, as the patient had not been fully compliant with the treatment.

The rarity of oesophageal GISTs explains the lack of clear recommendations regarding their optimal surgical management. As oesophageal segmental and wedge resections are not feasible, the surgical options include the high morbidity/mortality associated oesophagectomy and the much less invasive surgical tumour enucleation [8, 22–25]. The oncological outcome of these two procedures is similar, providing proper selection of patients (enucleation indicated for smaller tumours and with no evidence of mucosal ulceration) [8, 22–25]. An alternative approach for lower oesophageal GISTs is the Merendino procedure, which is utilized in our hospital providing that the tumour is accessible transhiatally [26]. This technique bears a low morbidity and mortality due to the avoidance of thoracotomy, provides a good functional outcome, and can also be applied to patients with tumours with mucosal ulceration (unpublished observations). Although our patient's comorbidities were favourable for a less invasive approach, both enucleation and Merendino procedure were not feasible. On one hand, enucleation was rejected because of the mucosal ulceration, and on the other hand, Merendino procedure in a patient with a high upper margin tumour would increase the possibility of an incomplete transhiatal tumour resection. Under these circumstances, we proceeded to a laparoscopic-assisted Ivor-Lewis oesophagectomy.

## 4. Conclusions

In the absence of clear guidelines for the optimal treatment of oesophageal GISTs, preoperative administration of imatinib in an attempt to downsizing, particularly in patients with large tumours, presented as an attractive approach aiming to (1) decrease the risk of intraoperative tumour rupture, thus achieving a better oncological outcome, and (2) reduce the extent of surgery and therefore the risks of perioperative morbidity and mortality. Compliance with drug therapy is essential for the success of this approach.
